# Roles of Regulatory T Cell-Derived Extracellular Vesicles in Human Diseases

**DOI:** 10.3390/ijms231911206

**Published:** 2022-09-23

**Authors:** Can Lin, Jihua Guo, Rong Jia

**Affiliations:** 1 The State Key Laboratory Breeding Base of Basic Science of Stomatology (Hubei-MOST) & Key Laboratory of Oral Biomedicine Ministry of Education, School & Hospital of Stomatology, Wuhan University, Wuhan 430079, China; 2 Department of Endodontics, School & Hospital of Stomatology, Wuhan University, Wuhan 430079, China

**Keywords:** regulatory T cells, extracellular vesicles, treatment, diagnosis, inflammation, immunotherapy

## Abstract

Regulatory T (Treg) cells play crucial roles in maintaining immune self-tolerance and immune homeostasis, and closely associated with many human diseases. Recently, Treg cells-derived extracellular vesicles (Treg-EVs) have been demonstrated as a novel cell-contact independent inhibitory mechanism of Treg cells. Treg-EVs contain many specific biological molecules, which are delivered to target cells and modulate immune responses by inhibiting T cell proliferation, inducing T cell apoptosis, and changing the cytokine expression profiles of target cells. The abnormal quantity or function of Treg-EVs is associated with several types of human diseases or conditions, such as transplant rejection, inflammatory diseases, autoimmune diseases, and cancers. Treg-EVs are promising novel potential targets for disease diagnosis, therapy, and drug transport. Moreover, Treg-EVs possess distinct advantages over Treg cell-based immunotherapies. However, the therapeutic potential of Treg-EVs is limited by some factors, such as the standardized protocol for isolation and purification, large scale production, and drug loading efficiency. In this review, we systematically describe the structure, components, functions, and basic mechanisms of action of Treg-EVs and discuss the emerging roles in pathogenesis and the potential application of Treg-EVs in human diseases.

## 1. Introduction

Regulatory T (Treg) cells are a special subpopulation of CD4^+^ T cells and are considered a vital regulator in maintaining immunological self-tolerance and immune homeostasis [1,2]. They are characterized by high expression of CD25 (cytokine IL-2 receptor alpha chain) and specific expression of transcription factor forkhead box protein P3 (FOXP3) [3,4,5,6,7]. Treg cells mainly develop in thymus-derived (tTreg cells or natural Treg cells, nTreg cells), but some Treg cells develop in the periphery (pTreg cells) [8]. Treg cells are involved in a variety of diseases, including autoimmune diseases, inflammatory diseases, transplantation rejection, and tumors [9]. Treg-mediated suppression can be attributed to contact-dependent or contact-independent mechanisms, such as expressing inhibitory receptors like cytotoxic T lymphocyte protein-4 (CTLA-4), CD39, and CD73 (Ecto-5-nucleotide enzyme), expressing perforin and granzyme B to kill target cells directly, the consumption of IL-2, and production of immunosuppressive cytokines (IL-10, IL-35, and TGF-β) [10,11,12,13,14]. Interestingly, numerous recent studies have demonstrated that Treg cells can release extracellular vesicles (EVs) to regulate target cells without direct contact with them [15,16,17,18].

Extracellular vesicles (EVs) are secreted by cells to the extracellular environment [19] and play a crucial role in intercellular communication by serving as vehicles for the transport of proteins, lipids, and nucleic acids [20]. EVs can be secreted by almost any cell type, including malignant cells and immune cells [21]. These vesicles play roles in antigen presentation, immune regulation, and signal transduction through autocrine and paracrine pathways, which are intimately related to the types of cells that release them [22]. According to vesicles’ sizes and production mechanisms, EVs can be divided into exosomes, micro-vesicles, and apoptotic bodies. Despite the mode of biogenesis being different, extracellular vesicle subtypes display a similar appearance, overlapping size, and standard composition [19]. The International Society for Extracellular Vesicles (ISEV) guidelines recommend using the operational terms for EV subtypes to replace the traditional classification [23]. In this review, we mainly use the general term “extracellular vesicles” based on the guidelines of MISEV2018.

Treg cells-derived extracellular vesicles (Treg-EVs) maintain self-immune tolerance and regulate immune responses by expressing specific molecules and delivering cargoes as a novel contact-independent mechanism of Treg cells. As Treg cells play an increasingly important role in immunity, research on Treg-EVs continues to deepen. In this review, we systematically describe the structure, components, and functions of Treg-EVs and discuss the possible use of Treg-EVs in human diseases.

## 2. The Structure and Composition of Extracellular Vesicles from Treg Cells

Extracellular vesicles are divided into exosomes, micro-vesicles, and apoptotic bodies. Exosomes are small membrane vesicles of 50 nm to 150 nm in diameter, produced by sprouting inward from the restricted membrane of endosomes to form a multivesicular body (MVB), then fused with the plasma membrane and released into the extracellular space. Micro-vesicles are directly generated from the plasma membrane, with diameters ranging from 150 nm to 1000 nm. Apoptotic bodies are only released from the surface of apoptotic cell during cell death, measuring 1–5 µm [24].

**Figure 1:**
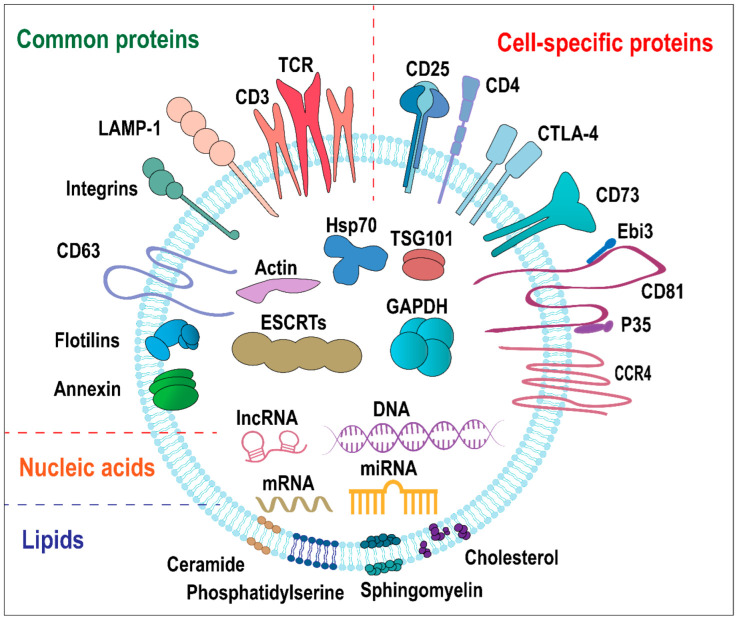
Schematic diagram of the structure and components of Treg-EVs. Treg-EVs are lipid bilayer sacs containing proteins, nucleic acids, and lipids. Treg-EVs proteins mainly include common proteins and cell-specific proteins. Common proteins are divided into membrane proteins (e.g., TCR/CD3, flotillin, annexins, CD63, integrin, and LAMP-1) and intracellular proteins (e.g., HSP70, ESCRT, TSG101, GAPDH, and Actin). Cell-specific proteins are associated with Treg cell surface proteins (e.g., CD4, CD25, CTLA-4, CD73, CCR4, and CD81 co-localized with IL-35 heterodimers). Nucleic acids in Treg-EVs include DNA, mRNA, miRNA, and lncRNA. The lipids in Treg-EVs include cholesterol, sphingolipids, and phospholipids, and ceramide.

Extracellular vesicles from different cells have similar elemental compositions and structures. Extracellular vesicles from Treg cells are also lipid bilayer sacs containing proteins, nucleic acids, and others [25]. However, Treg-EVs contain cell type-specific biological molecules, mainly related to the immune function of Treg cells. Treg-EVs modulate immune responses by expressing specific molecules and delivering unique genetic materials. We will focus on the three types of major components of Treg-EVs, namely, protein, nucleic acid, and lipid in the following contents (Table 1 and Figure 1).

### 2.1. Protein

Proteins carried by Treg-EVs have always been the focus of people’s attention. In general, EV proteins can be associated with the lumen, including those in the limiting membrane, or sometimes related to the luminal surface of EVs [19]. EVs are composed of lipid bilayer membranes. The transmembrane proteins expose their extracellular domains to the extracellular space of EVs, which is essential for EVs to interact with their respective receptors. Notably, soluble cytosolic proteins can be encapsulated in EVs as cargoes, which are protected from external proteases [16].

#### 2.1.1. Common EV Proteins

Like EVs derived from other cell types, Treg-EVs contain many common EV proteins, which are considered as universal markers. These rich protein components play essential roles in EVs biogenesis and secretion. The common proteins on the surface of extracellular vesicles include membrane transporters and fusion proteins (e.g., flotillin and annexins), tetraspanins (e.g., CD63 and CD81), cell adhesion-related proteins (e.g., integrin), lysosomal-related membrane proteins (e.g., LAMP), and some glycoproteins. The common proteins located inside extracellular vesicles include heat shock protein (e.g., HSP70), poly-vesicle biosynthesis-related proteins, endosomal sorting complex for transport (ESCRT) and its auxiliary protein components (e.g., TSG101), and cytoskeletal proteins (e.g., actin, tubulin). There are also some common metabolic enzymes (e.g., GAPDH), signaling proteins, and carrier proteins [19,30]. At present, the common EV-proteins CD63 and CD81, together with electron microscopy results, are used to identify and isolate Treg-EVs.

#### 2.1.2. Treg-EVs Specific Proteins

More importantly, Treg-EVs consist of some unique proteins, which can be used to distinguish them from other cells-derived EVs and are directly related to their function. Murine CD4^+^ CD25^+^Foxp3^+^ cells-derived EVs express high level of CD25 and low level of cytotoxic T lymphocyte-associated protein 4 (CTLA-4), as well as CD4^+^ T cell-type specific proteins, such as CD4, CD2, and MHC class I [16]. Human CD4^+^CD25^high^CD127^low^ Treg cells-derived EVs express CD25 and homing receptor CCR4, as well as low levels of CD4 and CTLA-4, without Fas-ligand [26]. CTLA-4, an immune checkpoint protein and a transmembrane protein highly expressed on Treg cells, is involved in a critical mechanism of Treg-mediated suppression [31]. However, the expression of CTLA-4 detected was too low to mediate significant suppression in both mouse and human Treg-EVs [16].

Transmembrane proteins CD73/CD39 on Treg-EVs can facilitate suppression through adenosine production. Murine Treg-EVs have a high expression of CD73, but human Treg-EVs express CD39 without CD73 [16,26]. The transmembrane proteins CD39 and CD73, as ectonucleotidases present on Treg cells, work together to stepwise hydrolyze extracellular ATP to immunosuppressive adenosine (ADO) [32]. CD39 catalyzes the production of adenosine monophosphate (AMP) from ATP, which is further hydrolyzed to adenosine by CD73. Adenosine binds to the adenosine receptor (A2AR) to activate related signaling pathways and mediate inhibitory effects [33]. CD73 is now considered the most critical molecule in murine Treg-EVs-mediated inhibition, the lack of CD73 on Treg-EVs reverses its inhibitory properties [16]. Human Treg-EVs also inhibited the proliferation of effector T cells (Teff), which may be related to the expression of CD39, but it is currently unclear [26]. Interestingly, Neuropilin-1 (Nrp1) expression is required for the expression of CD73 in Treg-EVs. Nrp1 is expressed on the membrane of Treg cells and contributes to maintain their phenotype and suppressive function [34,35]. Treg-EVs without Nrp1 failed to inhibit conventional T cell proliferation, and to facilitate skin transplantation tolerance. Nrp1 knockout led to less expression of CD73 in Treg-EVs [36].

Interleukin-35 (IL-35) on the surface of Treg-EVs can promote immune tolerance in a novel way [27]. IL-35, a heterodimeric cytokine mainly produced by Treg cells, is composed of Epstein–Barr virus-induced gene 3 beta-chain (Ebi3) and IL-12 alpha chain (p35) subunits [37]. Unlike other soluble cytokines, IL-35 is associated with the tetraspanin CD81 as the component on the surface of Treg-EVs. IL-35-coated EVs deliver IL-35 to the surface of many target B and T cells and induce secondary suppression. This special cytokine delivery method is a potential immune suppression mechanism mediated by Treg-EVs [27].

Altogether, the common proteins CD63 and CD81 can be used to identify and isolate extracellular vesicles. Cell-specific proteins are closely related to the specific functions of Treg-EVs.

### 2.2. Nucleic Acids

The first reported nucleic acid in extracellular vesicles was the microRNA (miRNA) and mRNA in 2007 [38]. Later, other types of nucleic acids were also discovered in extracellular vesicles, including DNA (mitochondrial DNA, single-stranded DNA, double-stranded DNA) [39], rRNAs [40], miRNAs [41], lncRNA [42], Y-RNA [43], snoRNA, and piRNA [44]. Up to now, the research interest of nucleic acids in Treg-EVs has mainly focused on microRNA.

MicroRNA is a family of small non-coding RNA of 21~25 nucleotides (nt) in length, which mainly negatively regulates gene expression in a sequence-specific manner. Specific miRNAs are selectively sorted into EVs, so the miRNA repertoires of EVs are usually different from that of parental cells [45,46]. Treg-EVs have been widely reported to transfer miRNAs to target cells and regulate cell functions. For example, murine Foxp3^+^ Treg cells-derived EVs preferentially transfer miR-155, let-7b, and let-7d to T helper 1 (Th1) cells, and let-7d can significantly inhibit Th1 cell proliferation and IFN-γ secretion [15]. The miRNA profiles of human Treg cells-derived EVs are significantly different from EVs released by other T cell subtypes. Compared with EVs derived from Th1 and Th17 cells, Treg-EVs are characterized by increased levels of miR-150-5p, miR-146a-5p, and miR-21-5p and decreased levels of miR-155-5p, miR-106a-5p, and miR-19a-3p [28].

Treg-EVs can also transfer miRNAs to dendritic cells (DCs) and modulate DCs function. The Treg cells generated by stimulation of C57BL/6 Foxp3^+^ cells with allogeneic BALB/c DCs produce EVs expressing low-level miR-384-5p and high levels of miR-142-3p and miR-150-5p, compared with CD4^+^Foxp3^-^ cells. Treg-EVs transfer miR-150-5p and miR-142-3p to DCs. These miRNAs induce a ‘tolerogenic’ phenotype by changing DCs cytokine profiles [29]. Therefore, specific miRNAs are enriched in Treg-EVs and transferred to corresponding target cells to play an immunomodulatory role.

### 2.3. Lipids

Lipids play an essential role in the process of EVs budding from the plasma membrane or endosomal membrane to form vesicles. Most lipids in EVs are cholesterol, sphingolipids, and phospholipids [45]. However, the lipid composition of EVs remains poorly explored in Treg-EVs, as well as in the EVs derived from other cell types.

The lipid composition of EVs is closely related to the types of cells from which they originate. However, due to the special way of production, the components of lipids in EVs are different from those in the original cell membrane. The common lipids in EVs include cholesterol (CHOL), diacylglycerol (DAG), sphingomyelin (SM), ceramide (Cer), phospholipids, phosphatidylcholine (PC), phosphatidylserine (PS), phosphatidylethanolamine (PE), phosphatidylinositol (PI), and glycerophospholipids. The proportion of SM, PS, PI, Cer, and CHOL in EVs is higher than the original cells. On the contrary, the proportion of PC in EVs is lower than the source cells [47,48]. The specificity of the lipid component of Treg EVs needs further exploration.

Lipids in EVs are essential for the formation and release of EVs, for the stability of vesicles during transport, and for facilitating the binding with target cells [49]. EVs may contain some bioactive lipids and enzymes responsible for synthesizing these bioactive lipids, which may affect the behavior of target cells. Through EVs-mediated lipid transfer, EVs serve as carriers to release lipids synthesized by derived cells and induce acceptor cells to produce lipids [50]. Thus far, there are few studies on the components and roles of lipids in Treg-EVs. Further research on Treg-EVs may reveal novel functions of lipids in Treg-EVs.

## 3. The Interaction between Treg-EVs and Target Cells

EVs mediate signal transduction and molecular transfer via several mechanisms and induce physiological changes in acceptor cells. In some cases, EVs stay on the surface of target cells and induce signal transduction by binding to specific receptors presented in the plasma membrane. In other cases, EVs are endocytosed into target cells or directly fused with the plasma membrane of target cells, thereby delivering their contents into the cytosol and then modifying or reprogramming the acceptor cells. EVs are internalized by multiple pathways, and fusion with lysosomes results in the degradation of extracellular vesicles and recycling of their contents. The direct fusion of extracellular vesicles with the membrane of recipient cells can release its contents into the cytoplasm of recipient cells and enables the exchange of transmembrane proteins and lipids [19,51]. The interaction between Treg-EVs and their target cells has significant effects on the functions of Treg-EVs in target cells. However, the mode of vesicle interaction with the cell surface and the mechanisms that mediate the transfer of extracellular vesicle cargoes are not fully unraveled, depending on the origin and specific composition of extracellular vesicle and the identity of the acceptor cell.

## 4. The Cellular and Molecular Functions of Treg-EVs

**Figure 2:**
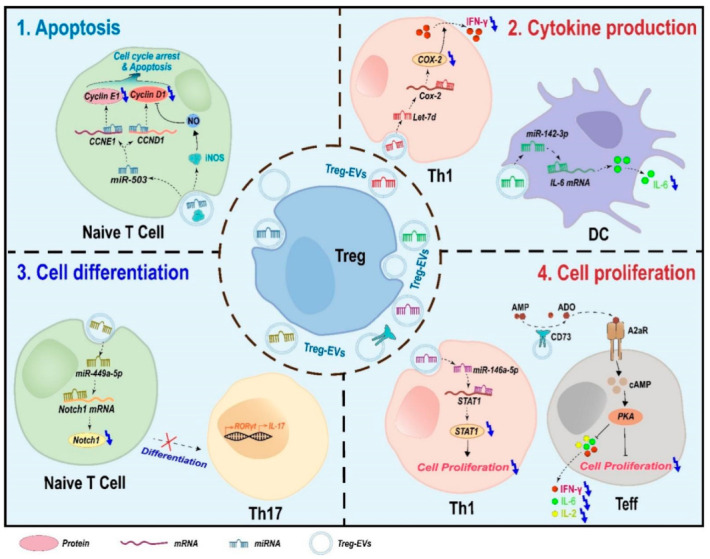
Schematic model for the suppressive mechanisms of Treg-EVs. Treg-EVs affect a variety of physiological processes including cell proliferation, apoptosis, cytokine production, and cell differentiation.

Treg cells can participate in immune regulation through releasing extracellular vesicles in a contact-independent manner. Treg-EVs possess the capacity to modulate immune responses by miRNAs-induced gene silencing, the activity of surface proteins, and the transmission of enzymes. In different target cells, Treg-EVs affect a variety of physiological processes, including cell proliferation, apoptosis, and cytokine production (Figure 2).

### 4.1. Apoptosis

Treg-EVs can induce apoptosis in conventional T cells [52]. The EVs derived from some Treg cells can transfer miR-503 and iNOS enzyme to naïve T cells, block their cell cycle progression and induce apoptosis [53]. MiR-503 induces G1 cell cycle arrest by reducing the expression levels of cyclin E and cyclin D1 proteins in target cells [54,55]. iNOS enzyme can catalyze NO production and induce the downregulation of cyclin D1 and cell cycle arrest [56,57]. However, similar to cell proliferation, Treg-EVs may inhibit the apoptosis of some specific types of cells. In immortalized murine colonic epithelial cell line YAMC cells, Treg-EVs can inhibit apoptosis by transferring mir-195a-3p, which negatively regulates the apoptosis-related protein expression [58]. Interestingly, Treg-EVs show the indirect anti-apoptotic effects in vivo. Treg-EVs can inhibit apoptosis of myocardial cells in acute myocardial infarction mouse model by promoting M2 polarization of macrophage in myocardial tissues [59]. Therefore, the effects of Treg-EVs on cell apoptosis and proliferation depend on cell type.

### 4.2. Cytokine Production

Treg-EVs can significantly change the cytokine expression profile of target cells. In Teff cell, CD73 expressed on Treg-EVs inhibited the production of cytokines including IL-2, IL-6, and IFN-γ in a dose-dependent manner [16]. Moreover, Treg-EVs mainly transfer miRNAs to regulate cytokine production of target cells. Human Treg-EVs could increase the levels of IL-4 and IL-10 and decrease the levels of IL-6, IL-2, and IFN-γ in Teff cells by transferring miR-146a-5 and miR-150-5p [26]. Treg-EVs also preferentially package and transfer miRNA let-7d to Th1 cells and then suppress IFN-γ secretion by repressing Cox-2 expression [15]. In addition, Treg-EVs can transfer miR-142-3p to DCs and decrease IL-6 because the 3′ UTR of IL-6 is targeted by miR-142-3p [29].

### 4.3. Cell Differentiation

Treg-EVs can regulate target cell differentiation. EVs derived from TGF-β-induced CD4^+^ CD25^+^ Treg cells (iTreg) showed a significantly higher miR-449a-5p expression level than those from non-induced cells. iTreg-EVs inhibited Th17 differentiation by targeting the Notch1 pathway of T cells by transferring miR-449a-5 [60].

### 4.4. Cell Proliferation

Treg-EVs can inhibit cell proliferation via at least two ways. First, Treg-EVs inhibit target cell proliferation via surface proteins. Treg-EVs can suppress effector T (Teff) cell proliferation by CD73/CD39. CD73 (ecto-5′-nucleotidase) had been indicated to be the vital surface molecule of Treg cells-mediated inhibitory function. CD73 converts AMP to anti-inflammatory adenosine, which interacts with adenosine receptors A2aR expressed on effector T cells and then activates the intracellular cAMP pathway to inhibit cell proliferation [16]. CD39 expressed on Treg-EVs may inhibit Teff cell proliferation by the same mechanism [26]. Second, Treg-EVs can transfer miRNAs to target cells and suppress cell proliferation. For example, Human Treg-EVs inhibit CD4^+^ T cell proliferation by delivering miR-146a-5p, which down-modulates critical genes IRAK2 and STAT1 necessary for T cell proliferation [28]. In addition, Treg-EVs transfer let-7d to Th1 cell and inhibit cell proliferation [15]. However, Treg-EVs may promote cell proliferation in some specific types of cell, such as immortalized murine colonic epithelial cell line YAMC cells [58].

## 5. The Roles of Treg-EVs in Human Diseases

Increasing evidence demonstrates that Treg cells play essential roles in various human diseases and conditions. The abnormal quantity or function of Treg-EVs is one of the possible mechanisms mediating Treg cell-associated diseases. Treg-EVs are involved in suppressing transplant rejection, inhibiting inflammatory diseases, and regulating autoimmune diseases. Currently, Treg-EVs are mainly obtained from isolated Treg cells, but differences in Treg cell sources and methods of Treg cells, and EVs isolation could lead to the heterogeneity of isolated Treg-EVs in studies (Table 2).

### 5.1. Transplantation Rejection

Recently, the roles of Treg-EVs in transplant rejection have significantly attracted the attention of researchers. At present, solid organ transplantation is still the first choice for treating end-stage organ failure. However, chronic immune rejection significantly limits the survival rate of the transplant. Patients have to take immunosuppressive drugs to prevent rejection in the long term, which often causes severe side effects. T regulatory cell-mediated transplantation tolerance is considered an attractive novel therapeutic strategy. Treg cells can reduce transplantation rejection by mediating immune suppression and maintaining immune tolerance [62,63]. Similar to Treg cells, Treg-EVs can also prevent transplantation rejection and prolong the survival time of transplant.

#### 5.1.1. Kidney Transplantation

Treg-EVs, especially exosomes with a size of 30–100 nm, could inhibit T cell proliferation, delay acute rejection, and then significantly prolong the survival time of allografts in a rat kidney transplantation model [18]. Similarly, Treg-EVs could also prolong the survival time of kidney transplantation in a rat model of kidney allograft by inhibiting T cell proliferation [53].

#### 5.1.2. Liver Transplantation

In a rat orthotopic liver transplantation (OLT) model, the injection of mouse natural CD4^+^ CD25^+^ Treg-EVs can significantly prolong the survival time of animals. Mechanically, Treg-EVs induced the cell cycle arrest in CD8^+^ cytotoxic T lymphocytes (CTLs) and then inhibited proliferation and cytotoxic activity by reducing the expression of perforin and IFN-γ, which play a crucial role in immune rejection [61].

#### 5.1.3. Skin Transplantation

In a mouse model of humanized skin transplantation, human Treg-EVs prevented alloimmune mediated human skin allograft damage and prolonged skin allograft survival by limiting immune cell infiltration. Mechanically, Treg-EVs modified the cytokine production of Teff, such as reducing IL-6 while increasing IL-4 and IL-10 [26]. Blocking IL-6 production and IL-6 signaling pathway [64,65] and increasing IL-4 level [66] can promote transplantation tolerance.

### 5.2. Autoimmune Diseases

Autoimmune diseases can be attributed to the immune responses to self-antigens resulting in damage or dysfunction of tissues, which are often accompanied by abnormal quantity and/or function of Treg cells [67]. Abnormal Treg-EVs are associated with the pathogenesis of various autoimmune diseases.

#### 5.2.1. Psoriasis

Psoriasis is a chronic, immune-mediated disorder manifesting in the skin and joints [68]. Treg cells are dysfunctional in most patients with psoriasis and play an important role in psoriasis pathogenesis. Treg-EVs may be associated with disease progression. The expression of multiple miRNAs derived from T cells was significantly upregulated in the sera of psoriasis patients compared with healthy donors. Interestingly, after treatment with etanercept (a tumor necrosis factor α (TNF-α) inhibitor biologic), the expression of miR-146a-5p enriched in Treg-EVs was increased, unlike other miRNAs that returned to normal levels. However, there is no direct evidence that miRNA changes in serum are associated with Treg-EVs. Therefore, the values of miRNAs derived from Treg-EVs in psoriasis need more extensive studies [28].

#### 5.2.2. Multiple Sclerosis (MS)

Multiple sclerosis is an autoimmune disease characterized by axonal degeneration of the central nervous system [52,69]. The defects in the Treg function have been considered one of the possible mechanisms leading to MS [70,71]. Compared with normal human Treg-EVs, the ability of extracellular vesicles from MS patients to inhibit the proliferation and induce apoptosis of conventional T cells (Tconv) was significantly reduced. Mechanically, the dysregulation of immunomodulatory molecules related Treg-EVs might contribute to insufficient Treg cell activity in MS patients [52].

#### 5.2.3. Rheumatoid Arthritis (RA)

Rheumatoid arthritis is an autoimmune disease characterized by synovial hyperplasia and irreversible destruction of articular cartilage [72]. Th17 cell expansion and impaired Treg function are associated with disease progression [73]. TGF-β-induced Treg cells-derived EVs (iTreg-EVs) preferentially localize to pathological joints and delay the occurrence of RA in a mouse collagen-induced RA experimental model. In this model, iTreg-EVs target the expression of the inflammatory gene Notch1 by transferring miR-449a-5p [60,74]. Notch signaling is involved in T cell development and differentiation, and inhibition of the Notch1 pathway could inhibit Th1 and Th17 cells, while promoting Treg cells. By transferring miR-449a-5p, iTreg-EVs reversed Th17/Treg imbalance to prevent disease progression and relieve RA symptoms [75,76].

### 5.3. Inflammation

Treg cells play key roles in suppressing inflammation, partially by releasing extracellular vesicles. Treg-EVs showed impressive roles in suppressing intestinal inflammation in animal models. Targeted inhibition of Th1 cell proliferation and IFN production by Treg-EVs is a possible mechanism to suppress inflammation. Let-7d deficient murine Treg-EVs failed to prevent colitis compared with wild-type Treg-EVs. The lack of Dicer (required for miRNA maturation) and Rab27 (required for vesicle release) or impaired transport of let-7d abolished the ability of Treg cells to suppress inflammation [15]. Let-7d of Treg-EVs targeting Cox-2 prevented Th1 cell-mediated intestinal inflammation [15,77].

In addition, Treg-EVs alleviate inflammatory bowel disease (IBD) by transferring miRNA to intestinal epithelial cells. IBD is a non-specific intestinal inflammatory disease with intestinal epithelial barrier dysfunction. In a dextran sodium sulfate (DSS)-induced IBD mouse model, Treg-EVs promoted the reparative process of intestinal epithelial barrier damage. Mechanically, Treg-EVs transferred miR-195a-3p to the intestinal epithelial cells, which directly targeted the mRNA of CASP12 gene, and negatively regulated the expression of Caspase 12, a pro-apoptotic protein. Therefore, Treg-EVs can promote intestinal epithelial cell proliferation and inhibit apoptosis and then reduce IBD symptoms [58].

### 5.4. Cancers

Treg-EVs may promote tumorigenesis via two ways. First, Treg-EVs inhibit cell proliferation of effector T cells [26] and cytokine production [15]. Moreover, Treg-EVs derived from natural CD8^+^ CD25^+^ regulatory T cells significantly inhibited DC-induced cytotoxic T lymphocyte responses and anti-tumor immunity in a mouse B16 melanoma model [17]. Second, the ability of Treg-EVs to promote the proliferation of immortalized epithelial cells [58] may also contribute to tumorigenesis. However, more studies are required to reveal the roles and mechanisms of Treg-EVs in tumorigenesis.

### 5.5. Other Diseases

Acute myocardial infarction (AMI) is an ischemic heart disease, and Treg cells can suppress the inflammatory response caused by myocardial infarction and promote pathological cardiac remodeling [78]. In an AMI mouse model, Treg-EVs reduced myocardial infarct size and myocyte cell apoptosis. The expression of iNOS (a marker of M1 macrophages), IL-1β, and TNF-α was notably suppressed in myocardial tissue treated by Treg-EVs. In contrast, Arg-1 (a marker of M2 macrophages) and TGF-β were significantly up-regulated. Interestingly, the depletion of mouse endogenous macrophages abolished the effects of Treg-EVs on AMI [59], suggesting that Treg-EVs may ameliorate AMI by promoting the polarization of macrophages to M2 style.

## 6. Application of Treg-EVs in Human Diseases

Based on the current research, infusion of Treg-EVs, as a new cell-free therapy, has excellent potential in disease diagnosis, inhibition of transplantation rejection, and even drug transport. At present, there are still many problems to be solved, but Treg cell-derived EVs have a bright prospect in the treatment of human disease. Compared with infused Treg cells, Treg-EVs are cell-free, easily enter specific tissues, and have no risk of Treg phenotypic conversion.

### 6.1. Transplantation and Autoimmune Diseases

Treg-EVs are a promising tool to treat immune-related diseases. Treg cells have been applied in the phase I/II clinical trials of patients with organ transplantation such as kidney transplantation (NCT02129881) [79] and liver transplantation (NCT02166177), as well as patients with autoimmune diseases such as autoimmune hepatitis (NCT02704338). However, the inflammatory environment may lead to human Treg cell instability and the phenotypic conversion of Treg cells into helper T cells (Th) such as Th1, Th2, and Th17 cells [80].

Like Treg cells, Treg-EVs may regulate immune responses, inhibit transplant rejection, and promote tissue regeneration. Recently, many types of EVs from mesenchymal stem cells (MSC), endothelial colony-forming cells (ECFC), neural stem cells (NSC), and other sources in regenerative therapy [81] have been used in pre-clinical and clinical studies. Therefore, Treg-EVs may be used to replace Treg cells for the treatment of inflammatory diseases directly. However, similar to other EVs, some technical issues must be solved before clinical application. It is a big challenge to isolate a large number of clinical-grade quality Treg-EVs under GMP conditions. Meanwhile, the technologies for the clinical-scale separation and in vitro expansion of Treg cells are also under development [81,82].

### 6.2. Biomarkers for Disease Diagnosis

Treg-EVs may be used as new and potential biomarkers for disease diagnosis. EVs are released by virtually all cell types in the body and have been widely found in various biofluids, including plasma [83], semen [84], urine [85], tumor effusions [86], and so on. More importantly, EVs express cell type-specific markers. The composition of EVs can reflect the physiological and pathological changes of cells or tissues from which EVs are derived, making the application of EVs possible as biomarkers for diagnosing diseases. The levels of EVs and the changes in their components such as proteins, miRNAs, and mRNAs can be used as diagnostic markers, closely correlated with the disease’s occurrence, development, and prognosis. The application of EVs as biomarkers in tumors [87], liver diseases [88], Parkinson’s disease [89], and other diseases has entered clinical research and has a large number of reviews.

As early as 2014, it was proposed that EVs derived from Treg cells could be used as a possible therapeutic and diagnostic tool in transplantation [82]. However, so far, Treg-EVs application has been limited in animal studies and not been used in clinical trials for several possible reasons, including the lack of standardized protocol for isolation and purification of Treg-EVs, as well as unified and standardized procedures for the analysis of Treg-EVs.

### 6.3. Delivery Media

As the messengers for intercellular communication, EVs can serve as the ideal carriers to transport therapeutic proteins, nucleic acids, and drugs to the target cells in clinical trials to treat diseases [90]. Treg-EVs can target a variety of cells, and the use of Treg-EVs for drug transport is a perfectly reasonable assumption after discovering EVs’ structural and functional features.

For example, the modified Treg-EVs can be used as a vehicle for the delivery of anti-VEGF antibodies (aV) to reduce choroidal neovascularization in both mouse and monkey models. The ocular neovascular disease is a progressive disease that can cause severe vision loss. Using antibodies targeting vascular endothelial growth factor (VEGF) is the most effective treatment. The rEXS–cL–aV, that is, Treg-EVs conjugated with aV using a peptide linker (cL), is a nanodrug. First, Treg-EVs enhance the delivery efficiency of aV. Conjugated aV transported by Treg-EVs accumulates in the neovascularization lesions. The ability of Treg-EVs to localize to neovascularization lesions, similar to the migration of Treg cells to sites of inflammation, might be attributed to the expression of chemokine receptors (such as CCR6) on Treg-EVs. Second, due to the slower elimination of Treg-EVs than that of soluble proteins, rEXS–cL–aV has prolonged the retention compared with aV alone. In addition, Treg-EVs mediate immunosuppression and synergize with aV to form a combination therapy. Treg-EVs contributed greatly to the high treatment efficacy of rEXS–cL–aV [91].

Obviously, in addition to the obstacles of Treg-EVs application mentioned above, the modification of EVs may generate additional difficulties. In fact, extracellular vesicle modification procedures may cause membrane damage, which may trigger the immune responses. Moreover, it is difficult to control the amounts of drugs loaded into EVs. In addition to simply mixing EVs with drugs to get hydrophobic compounds into EVs, commonly used methods include physical or chemical induction, which have their limitations. At present, studies have shown that the use of different routes of administration causes EVs to have different biodistribution patterns in the human body. Therefore, formulating an appropriate dosage regimen must also be considered [92]. Nonetheless, Treg-EVs as a drug-delivery system are a promising avenue for treating human diseases.

## 7. Conclusions and Remarks

In conclusion, Treg-EVs represent one of the key mechanisms of Treg cell-mediated suppression and are closely associated with human diseases. Treg-EVs carry many immunosuppressive molecules originated from parent Treg cells and repress the proliferation and function of target cells, especially T cells. Treg-EVs may be the promising novel targets for human disease treatment. Moreover, Treg-EVs can be used as biomarkers for disease diagnosis and drug delivery media.

Although encouraging results have been achieved with Treg-EVs, several aspects remain to be explored before Treg-EVs can be used for clinics. First, the roles and specific mechanisms of Treg-EVs in human diseases need further investigation. Second, standard operating procedures and large-scale production of Treg-EVs for clinical use remain to be explored. Third, it is necessary to improve the efficiency of drug loading in Treg-EVs and explore the route and dose of Treg-EVs. Therefore, future investigations should focus on combining basic research on Treg-EVs with emerging technologies to bring breakthroughs for the treatment.

## Figures and Tables

**Table 1:** Summary of specific cargo of Treg cells-derived extracellular vesicles.

Treg-EVs Source	Characterization Method	Size; Markers	Specific Cargo	Treg-EVs Function	Ref.
**Protein**					
Mouse natural Treg cells; murine Treg-cell line with self-specificity (Auto-Treg cells)	EM and flow cytometry	Mean 100 nm; LAMP-1/CD63 and CD81	Transmembrane proteins: CD4, CD2, MHC class I, high levels of CD73 and CD25, and low level of CTLA-4	CD73 contributes to Treg suppressive activity through adenosine production	[16]
Human natural Treg cells	EM, NTA, flow cytometry, ELISA, and Western blot	Mean 150 nm; CD63 and CD81	Transmembrane proteins: CD25, CD39, CCR4, low levels of CD4 and CTLA-4	Inhibiting T cell proliferation by unknown mechanism	[26]
IL-35-producing Treg cells	TEM, NTA, ELISA, and Western blot	50–200 nm; CD81	Transmembrane proteins: CD39, CD73, and IL-35 subunits (p35 and Ebi3)	Delivering IL-35 to the surface of B and T cells and induced secondary suppression	[27]
**MicroRNA**					
Mouse natural Treg cells	Flow cytometry, dynamic light scatter, and ELISA	20–100 nm; CD9, CD63, and CD81	miR-155, let-7b, and let-7d	Inhibiting Th1 cell proliferation and IFN-γ secretion by delivering let-7d	[15]
Human natural Treg cells	TEM, NTA, Western blot, and flow cytometry	Mean 140 nm; presence of clathrin and absence of calnexin	High levels of miR-150-5p, miR-146a-5p, and miR-21-5p, and low levels of miR-155-5p, miR-106a-5p, and miR-19a-3p	Inhibiting CD4^+^ T cell proliferation by delivering miR-146a-5p	[28]
Mouse Treg cells stimulated by dendritic cell	EM and NanoSight	Mean 100 nm; markers NA	Low levels of miR-384-5p and high levels of miR-142-3p and miR-150-5p	Changing DCs cytokine profiles by delivering miR-150-5p and miR-142-3p	[29]

Legend: EM, electron microscopy; NTA, nanoparticle tracking analysis; ELISA, enzyme linked immunosorbent assay; TEM, transmission electron microscopy.

**Table 2:** Heterogeneity of Treg cell-derived EVs in studies and their roles in human diseases and conditions.

Diseases and Conditions	Source of Tregs	Tregs Isolation Method	Treg-EVs Isolation Method	Effective Molecule	Roles of Treg-EVs	Reference
Kidney transplantation	Rat Lymphocytes	FACS	Ultracentrifugation (110,000× *g*) and 30% sucrose/D2O density cushion	Not determined	Prolonging kidney allograft survival	[18]
	Rat CD4^+^CD25^−^ regulatory T cells	FACS	Ultracentrifugation (100,000× *g*)	miR-503 and iNOS	Prolonging kidney allograft survival by inhibiting T cell proliferation	[53]
Liver transplantation	Mouse spleen lymphocytes	FACS	Ultracentrifugation (110,000× *g*) with filtration (0.22 μm)	Not determined	Prolonging liver allograft survival by suppressing CD8^+^ cytotoxic T lymphocyte proliferation	[61]
Skin transplantation	Human blood	RosetteSep kit and CD25 Microbeads kit	Ultracentrifugation (100,000× *g*) with filtration (0.22 μm) and ExoQuick-TC	Not determined	Prolonging skin allograft survival by modify T-effector cell cytokine production	[26]
Psoriasis	Human peripheral blood mononuclear cells	FACS	Ultracentrifugation (100,000× *g*), microfiltration (ExoMir Mini kit) and differential precipitation (ExoSpin kit)	Not determined	Associated with psoriasis pathogenesis	[28]
Multiple sclerosis (MS)	Human peripheral blood mononuclear cells	Dynabead Regulatory CD4^+^CD25^+^ T cell kit	Total Exosome Isolation kit	Not determined	Suppressing the proliferation of conventional T cells	[52]
Rheumatoid arthritis (RA)	TGF-β-induced Treg cells-derived EVs (iTreg-EVs)	FACS	EV isolation kits	miR-449a-5p	Reversing Th17/Treg imbalance to prevent RA progression	[60]
Intestinal inflammation	Mouse spleen	FACS	Ultracentrifugation (100,000× *g*) and ExoQuick solution	let-7d	Preventing colitis by inhibiting Th1 cell proliferation	[15]
	Mouse spleen mononuclear cells	Mini-MACS immunomagnetic separation system	Ultracentrifugation (100,000× *g*) with filtration (0.22 μm)	miR-195a-3p	Alleviating inflammatory bowel disease by promoting proliferation and inhibiting apoptosis of colonicepithelial cells	[58]
B16 melanoma	Mouse CD8^+^CD25^+^ regulatory T cells	MACS beads immunomagnetic separation system	Ultracentrifugation	Not determined	Suppressing cytotoxic T lymphocyte-mediated immunity against B16 melanoma	[17]
Acute myocardial infarction (AMI)	Mouse spleen	CD4^+^CD25^+^ regulatory T cell isolation kit	Total exosome isolation reagent kit	Not determined	Ameliorating AMI by promoting macrophage M2 polarization	[59]

## Data Availability

Not applicable.
